# Effects of Tenofovir on the Single-Dose Pharmacokinetics of Intravenous Morinidazole in Healthy Chinese Subjects

**DOI:** 10.1128/AAC.02067-19

**Published:** 2020-04-21

**Authors:** Guolan Wu, Wenling Tang, Duo Lv, Lihua Wu, Huili Zhou, Xi Yang, Yunliang Zheng, You Zhai, Jianzhong Shentu

**Affiliations:** a Research Center of Clinical Pharmacy, State Key Laboratory for Diagnosis and Treatment of Infectious Disease, First Affiliated Hospital, College of Medicine, Zhejiang University, Hangzhou, China; b Zhejiang Provincial Key Laboratory for Drug Evaluation and Clinical Research, First Affiliated Hospital, College of Medicine, Zhejiang University, Hangzhou, China; c College of Medicine, Zhejiang University, Hangzhou, China; d Department of Pharmacy, First Affiliated Hospital, College of Medicine, Zhejiang University, Hangzhou, China

**Keywords:** morinidazole, tenofovir, clinical pharmacokinetics, drug-drug interaction, organic anion transporter

## Abstract

The effects of multiple-dose administration of tenofovir disoproxil fumarate (TDF) on the pharmacokinetics of morinidazole (MOR) were compared in healthy subjects. MOR exposure was similar, with an area under the curve from 0 h to infinity (AUC_0-∞_) treatment ratio for MOR+TDF/MOR of 1.01 (90% confidence interval, 0.97 to 1.06). No relevant differences were observed regarding plasma exposure of metabolites. Renal clearances of MOR and its metabolites were not affected by TDF. No unexpected safety or tolerability issues were observed.

## Abstract (precis)

The effects of multiple-dose administration of tenofovir disoproxil fumarate (TDF) on the pharmacokinetics of morinidazole (MOR) were compared in healthy subjects. MOR exposure was similar, with an area under the curve from 0 h to infinity (AUC_0-∞_) treatment ratio for MOR+TDF/MOR of 1.01 (90% confidence interval, 0.97 to 1.06). No relevant differences were observed regarding plasma exposure of metabolites. Renal clearances of MOR and its metabolites were not affected by TDF.

## TEXT

Morinidazole is a new type of broad-spectrum nitroimidazole antimicrobial that exhibits greater activity and less toxicity than metronidazole (1, 2). It has been reported that morinidazole undergoes extensive metabolism, primarily via *N*-glucuronidation (yielding the *N*-glucuronide of *S*-morinidazole [M8-1] and of *R*-morinidazole [M8-2]) and *o*-sulfation (yielding the sulfate conjugate of morinidazole [M7]) (3). Morinidazole and its conjugates (M7, M8-1, and M8-2) are mainly excreted through urine (4). The main transporters involved in the renal secretion of drugs in humans include organic anion transporter (OAT) 1, OAT 3, and organic cation transporter (OCT) 2 (5–7). Morinidazole is not a substrate for OAT 1/3 or OCT 2, whereas M7, M8-1, and M8-2 are OAT 1/3 substrates (4). However, the contributions of renal uptake and efflux transporters in the active renal tubular secretion of morinidazole and its conjugated metabolites remain unclear. The competition or noncompetition inhibition of OAT transport function reduces renal clearance and causes bad clinical outcomes (8).

Tenofovir disoproxil fumarate (TDF) is an oral prodrug of tenofovir and a potent substrate of OATs, which are responsible for its tubular secretion into urine (9). TDF and morinidazole are likely to be used concomitantly for the treatment of mixed infections. Therefore, it was important to exclude the drug-drug interaction (DDI) potential when morinidazole was administered with TDF.

A prospective single-center, open-label crossover study was performed to assess the effect of tenofovir on the pharmacokinetics of morinidazole in healthy adults. The current study conformed to regulatory guidelines on the conduction of DDI studies (https://www.fda.gov/media/82734/download). The study protocol and informed consent documents were reviewed and approved by the Ethics Committee of the First Affiliated Hospital of Zhejiang University (approval no. 2017-EC-70). The Chinese Clinical Trial Registry number was ChiCTR-IIR-17012161.

**FIG 1:**
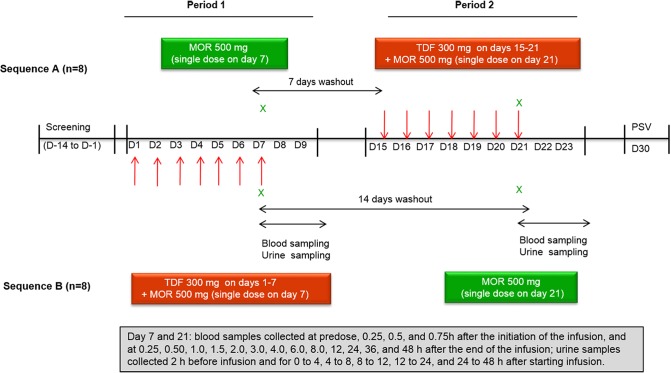
Study design.

The subjects were randomized into two treatment sequences, and each subject received two treatments, which included morinidazole alone and morinidazole in combination with TDF (Fig. 1). Sixteen healthy subjects (8 men and 8 women) were enrolled, but 1 woman withdrew from the study before initiation of period 1 because of herpesvirus infection. Fifteen subjects received 300 mg of TDF once daily for 6 days, 1 h before breakfast. They received the seventh TDF dose 1 h before the single 45-minute intravenous infusion of morinidazole. No breakfast was supplied, and no food was allowed for 4 h after administration of the drug on days 7 and 21. Blood and urine samples were collected at the specified time points (Fig. 1). Morinidazole, M2, M7, M8-1, and M8-2 were determined by liquid chromatography-tandem mass spectrometry. The main pharmacokinetic parameters were calculated by noncompartmental methods using WinNonlin 6.3 software.

**FIG 2:**
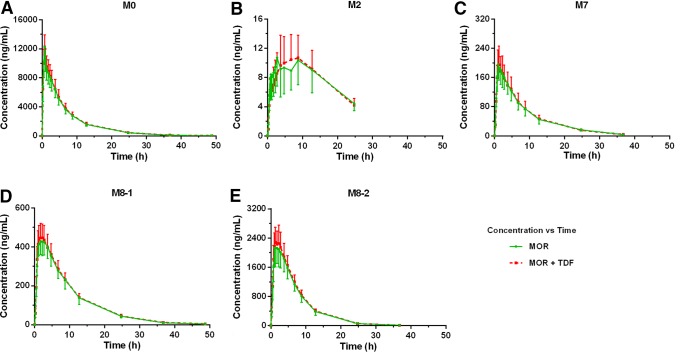
Mean plasma concentration-time proﬁles of morinidazole (MOR) (A), M2 (B), M7 (C), M8-1 (D), and M8-2 (E) after intravenous infusion of morinidazole 500 mg in healthy Chinese subjects with or without pretreatment with TDF. Data expressed as means ± standard deviation (*n *= 15). M0, morinidazole.

**FIG 3:**
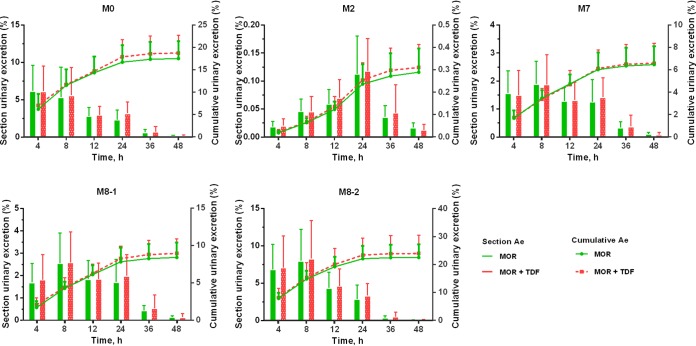
Urinary excretion of morinidazole (MOR) and its major metabolites after intravenous infusion of morinidazole 500 mg, with or without pretreatment with TDF, in healthy Chinese subjects. Data expressed as means ± standard deviation (*n *= 15). Ae, amount of drug excreted; M0, morinidazole.

The mean concentration-time profiles of morinidazole, M2, M7, M8-1, and M8-2 were not affected by TDF coadministration (Fig. 2, Table 1). Drug excretion rates and renal clearance (CL_R_) values were also unaffected by TDF coadministration (Fig. 3). The potential DDI risk of tenofovir on morinidazole major metabolites was also evaluated based on FDA guidelines (10). Consistent with previous studies, morinidazole was found to be the major circulating drug-related component, whereas the systemic exposures of M8-2, M7, and M8-1 were approximately 14.4%, 1.8%, and 4.0% of the parent drug, respectively (4, 11). The metabolic ratio of M2 (C_9_H_16_N_4_O_4_, the loss of C_2_H_2_ from morinidazole) in plasma and urine was similar in each group (Fig. 2 and 3). Exposure results for the primary endpoint area under the curve from 0 h to infinity (AUC_0–∞_) and other pharmacokinetic measures indicated that concomitant administration with TDF had no clinically relevant effect on the exposure of morinidazole and its major metabolites.

**TABLE 1:** Pharmacokinetic parameters of morinidazole and its major metabolites after intravenous infusion of morinidazole 500 mg in healthy subjects with or without treatment with TDF

Parameter^*a*^	Result (mean ± SD) of treatment with:		MOR+TDF:MOR (GLSM ratio [90% CI])
	MOR (*n* = 15)	MOR+TDF (*n* = 15)	
Morinidazole			
*C*_max_ (ng · ml^−1^)	12,514.09 ± 1,913.83	12,462.65 ± 1,459.58	1.00 (0.92–1.07)
AUC_0–_*_t_* (ng · h · ml^−1^)	76,590.73 ± 11,262.1	77,787.11 ± 11,918.50	1.01 (0.97–1.06)
AUC_0–∞_ (ng · h · ml^−1^)	76,830.62 ± 11,263.1	78,074.47 ± 11,928.26	1.01 (0.97–1.06)
*T*_max_ (h)^*b*^	0.75 (0.50–1.25)	0.75 (0.50–1.00)	
*t*_1/2_ (h)	5.95 ± 0.36	6.20 ± 0.35	
*V* (liters)	57.34 ± 11.23	58.87 ± 11.90	
CL (liters · h^−1^)	6.65 ± 1.05	6.56 ± 1.09	
CL_R_ (liters · h^−1^)	1.16 ± 0.26	1.22 ± 0.25	
M2			
*C*_max_ (ng · ml^−1^)	12.47 ± 3.69	11.45 ± 3.72	0.91 (0.82–0.99)
AUC_0–_*_t_* (ng · h · ml^−1^)	180.43 ± 75.40	183.96 ± 70.79	1.03 (0.88–1.18)
AUC_0–∞_ (ng · h · ml^−1^)	382.13 ± 202.48	294.32 ± 73.50	0.86 (0.56–1.18)
*T*_max_ (h)^*b*^	3.75 (0.75–12.75)	8.75 (3.75–12.75)	
*t*_1/2_ (h)	26.36 ± 28.42	10.99 ± 1.75	
*V* (liters)	43,181.69 ± 27,863.49	28,567.50 ± 9,595.22	
CL (liters · h^−1^)	1,541.17 ± 529.15	1,787.14 ± 433.47	
CL_R_ (liters · h^−1^)	7.73 ± 2.96	8.60 ± 3.79	
M7			
*C*_max_ (ng · ml^−1^)	208.42 ± 47.29	205.02 ± 49.55	1.00 (0.92–1.04)
AUC_0–_*_t_* (ng · h · ml^−1^)	1,798.51 ± 409.09	1,807.14 ± 401.58	1.00 (0.94–1.08)
AUC_0–∞_ (ng · h · ml^−1^)	1,842.16 ± 407.22	1,847.11 ± 399.83	1.00 (0.93–1.08)
*T*_max_ (h)^*b*^	1.00 (1.00–1.75)	1.25 (1.00–2.75)	
*t*_1/2_ (h)	6.44 ± 0.48	6.57 ± 0.43	
*V* (liters)	2,627.90 ± 583.84	2,668.14 ± 531.55	
CL (liters · h^−1^)	281.98 ± 52.99	280.73 ± 50.69	
CL_R_ (liters · h^−1^)	23.50 ± 4.34	24.04 ± 6.26	
M8-1			
*C*_max_ (ng · ml^−1^)	445.04 ± 61.59	465.54 ± 70.31	1.00 (0.99–110.6)
AUC_0–_*_t_* (ng · h · ml^−1^)	5,065.55 ± 807.37	5,176.49 ± 694.49	1.00 (0.98–1.08)
AUC_0–∞_ (ng · h · ml^−1^)	5,120.78 ± 800.43	5,235.34 ± 695.45	1.00 (0.98–1.08)
*T*_max_ (h)^*b*^	2.25 (1.00–3.75)	1.75 (1.00–2.75)	
*t*_1/2_ (h)	6.47 ± 0.55	6.55 ± 0.46	
*V* (liters)	932.14 ± 169.12	916.50 ± 138.01	
CL (liters · h^−1^)	99.80 ± 15.06	97.01 ± 12.33	
CL_R_ (liters · h^−1^)	13.86 ± 3.12	14.44 ± 3.42	
M8-2			
*C*_max_ (ng · ml^−1^)	2,248.67 ± 481.29	2,376.93 ± 473.02	1.00 (1.00–1.12)
AUC_0–_*_t_* (ng · h · ml^−1^)	18,203.89 ± 3,139.73	18,953.68 ± 2,511.84	1.00 (1.00–1.11)
AUC_0–∞_ (ng · h · ml^−1^)	18,466.67 ± 3,103.75	19,231.04 ± 2,532.43	1.00 (1.00–1.10)
*T*_max_ (h)^*b*^	1.30 (1.00–2.75)	1.75 (1.00–2.75)	
*t*_1/2_ (h)	4.19 ± 0.43	4.10 ± 0.29	
*V* (liters)	168.60 ± 34.40	156.64 ± 26.25	
CL (liters · h^−1^)	27.73 ± 4.23	26.24 ± 3.43	
CL_R_ (liters · h^−1^)	10.40 ± 2.41	10.48 ± 2.54	

a AUC_0–_*_t_*, area under the concentration-time curve from 0 h to the last sampling time; AUC_0–∞_, area under the concentration-time curve from 0 h to inﬁnity; CI, conﬁdence interval; CL, apparent clearance; CL_R_, renal clearance; *C*_max_, maximum observed concentration; GLSM, geometric least-squares mean; MOR, morinidazole; *V*, volume of distribution; TDF, tenofovir disoproxil fumarate; *T*_max_, time to *C*_max_; *t*_1/2_, terminal elimination-phase half-life.

b Data expressed as median (range).

There are several potential explanations for the finding that tenofovir did not cause a clinical DDI. First, the systemic concentration of tenofovir may not have been high enough to inhibit the transport of morinidazole-conjugated metabolites. Second, it may take ≥2 weeks of daily TDF administration to achieve the maximum level of inhibition. Third, other uptakes or efflux transporters, such as MRP4, might have led to competing inhibition and be involved in morinidazole and its metabolite renal excretion. Finally, merely the total drug plasma concentration was determined. Thus, plasma protein binding may have covered up a possible clinical DDI.

The pharmacokinetic properties of morinidazole were similar to those in previous studies (3, 11). The renal clearance of morinidazole and its conjugated metabolites in healthy volunteers was much higher than the values previously reported for patients with severe renal impairment (Table 1). Furthermore, the study conducted by Kong F et al. (12) clarified that accumulated uremic toxins may inhibit transporters, particularly OAT 3, leading to plasma exposure changes in relevant substrates. Thus, future investigations are needed to evaluate the effect of TDF administration on morinidazole pharmacokinetics in patients.

Adverse events (AEs), vital signs, pregnancy tests, clinical laboratory tests, and electrocardiograms (ECGs) were monitored to assess safety. Morinidazole alone and coadministered with TDF were well tolerated by the volunteers. As shown in Table 2, all AEs were transient and were grade 1 (mild) in severity. No serious AEs occurred during the study, and 15 patients were in good compliance with the protocol. No clinically significant changes in physical exams or ECGs were observed.

**TABLE 2:** Summary of adverse events during study period

Adverse event parameter^*a*^	Treatment		Total
	MOR	MOR+TDF	
No. (%) of subjects with ≥1 AE	5 (33.3%)	4 (26.7%)	9 (60.0%)
No. of AEs	10	6	16
No. (%) of subjects with ≥1 TEAE	3 (20.0%)	4 (33.3%)	7 (46.7%)
TEAE, total (*n*)	4	6	10
Leucopenia	1	0	
Neutropenia	1	0	
Urine protein positive	0	2	
Urinary occult blood test positive	0	1	
Fasting venous glucose increased	0	1	
Serum triglycerides increased	1	0	
Constipation	0	1	
Abdominal distension	0	1	
Fever	1	0	

a AE, adverse event; TEAE, treatment-emergent adverse event.

However, the current study has some limitations. First, the sample size was small, and the sample consisted of young and healthy subjects exposed to TDF for a short period in contrast to elderly patients with hepatic impairment. Second, tenofovir is not recommended by the FDA as an OAT 1/3 inhibitor. Therefore, further clinical studies are needed to evaluate the clinical DDI risk that involves OAT 1/3 between morinidazole and the recommended inhibitors. Although previous studies have shown no significant accumulation after multiple doses and no need for dose adjustment of TDF in patients with hepatic impairment (13–15), caution should be exercised when extrapolating these data for patients with abnormal liver function under long-term TDF treatment.

In conclusion, this study demonstrated that coadministration of the approved clinical dose of 300 mg of TDF has little effect on systemic exposure of morinidazole or its main metabolites at a single intravenous dose of 500 mg. Morinidazole and TDF, alone and combined, were well tolerated.
